# Machine-learning prediction of 3- and 5-year mortality in lymph-node-positive medullary thyroid carcinoma: a study based on the SEER database and external validation in a Chinese cohort

**DOI:** 10.3389/fonc.2026.1798296

**Published:** 2026-04-14

**Authors:** Shaojun Yang, Ruming Zhao

**Affiliations:** Department of Oncology, Zibo Municipal Hospital, Zibo, Shandong, China

**Keywords:** cancer-specific survival, lymph node metastasis, machine learning, medullary thyroid carcinoma, overall survival

## Abstract

**Background:**

Medullary thyroid carcinoma (MTC) carries a disproportionately high mortality among thyroid malignancies, and the risk is even greater once metastasis occurs; nevertheless, a dependable prognostic tool for lymph-node-positive MTC patients remains elusive. We aimed to derive and externally validate a machine learning model for predicting 3-year and 5-year overall survival (OS) and cancer-specific survival (CSS) in this high-risk population.

**Methods:**

Retrospective cohorts were assembled from the U.S. SEER database (n = 1,071) and Zibo Municipal Hospital (external validation, n = 198). After feature selection (Cox, Boruta, RFE), five algorithms (LightGBM, XGBoost, RF, MLP, KNN) were trained in 70% SEER data and tested in the remaining 30% and in the Chinese cohort. F1-score, MCC, sensitivity, specificity, AUC, calibration curve, and decision-curve analysis were evaluated; model explainability was assessed with SHAP.

**Results:**

In OS prediction, LightGBM achieved the highest AUC in both time horizons (SEER 3-year 0.833, 5-year 0.892; external 5-year 0.869), with superior accuracy. Calibration curves lay closest to the 45° diagonal, and decision-curve analysis demonstrated the greatest net benefit across clinically relevant risk thresholds. SHAP revealed the absence of surgery as the strongest adverse contributor for OS, followed by advanced age, larger tumour size, higher LNR, radiotherapy and chemotherapy demonstrated adverse effects. The same pattern emerges when predicting CSS. Based on these results, we developed an online calculator for predicting 3- and 5-year OS and CSS in patients with lymph-node-positive MTC.

**Conclusion:**

LightGBM model provides an accurate, well-calibrated, and clinically useful tool for estimating survival in lymph-node-positive MTC. In addition, the decision to undergo surgery is considered the most important factor in the survival of MTC patients.

## Introduction

Medullary thyroid carcinoma (MTC), a neuroendocrine malignancy arising from parafollicular C-cells, accounts for approximately 4% of all thyroid cancers. Although its incidence is low, MTC attracts considerable clinical attention due to its aggressive biological behavior and unfavourable prognosis (1, 2). The tumour frequently disseminates to regional lymph nodes and distant organs, and recurrence rates after supposedly curative surgery remain as high as 40-66% (3–5). While total thyroidectomy with compartment-oriented lymphadenectomy remains the cornerstone of therapy, conventional adjuvant radiotherapy or chemotherapy offers limited benefit in advanced disease. Consequently, patients who present with metastatic MTC face a near 50% disease-specific mortality and carry a heavy psychosocial and economic burden (6–9). There is therefore an urgent need to develop precise prognostic tools and individualized therapeutic strategies for this high-risk population.

Accumulating evidence has identified several conventional prognostic factors for MTC, including patient age, tumour stage, sex, primary tumour size, lymph-node metastasis, serum calcitonin level, somatic RET mutation subtype, initial treatment response, and the extent of thyroidectomy (3, 10–12). Nevertheless, data specifically addressing the outcomes of node-positive MTC remain scarce. Concurrently, the rapid evolution of machine learning methodologies, with their formidable capacity for high-dimensional data processing and pattern recognition, has markedly improved prognostication in oncology and has been successfully applied to thyroid cancers (13–16). To date, however, no rigorously developed machine-learning framework has been constructed to predict long-term survival in patients with lymph-node-positive MTC.

To fill these knowledge gaps, we constructed a large retrospective cohort of lymph-node-positive MTC patients by integrating the Surveillance, Epidemiology, and End Results (SEER) database with clinical data from Zibo Municipal Hospital. Demographic, pathologic, and treatment variables were extracted; by identifying the most accurate predictors and the optimal machine-learning model, we aimed to provide clinicians with a robust, evidence-based decision-support tool that can refine risk stratification and guide individualized treatment planning for this high-risk population.

## Methods

### Data source

Study data were extracted from the SEER database maintained by the U.S. National Cancer Institute (NCI). The registry aggregates cancer incidence and survival information from 18 population-based geographic regions, covering approximately 30% of the U.S. population, and constitutes the principal source of national cancer statistics (17, 18). Case listings were generated with SEER*Stat software (version 8.3.6; https://seer.cancer.gov/data/). For each patient, follow-up was calculated from the date of initial diagnosis to the date of last known contact or death; vital status and underlying cause of death were also provided (19). As SEER is a publicly available, de-identified database, institutional review board approval was not required.

External validation data were obtained from Zibo Municipal Hospital with approval from the Zibo Municipal Hospital Ethics Committee.

### Study population

Eligibility criteria were applied sequentially. First, we retained patients whose primary tumour was located in the thyroid (ICD-O-3 topography code C73.9; n = 240,557). We then excluded the following: (i) histologies other than MTC (n = 236,248); (ii) cases without pathologically confirmed regional lymph-node metastasis (n = 2,658); (iii) patients with multiple primary malignancies (n = 430), age ≤ 20 years (n = 43), or survival < 1 month from diagnosis (n = 26); and (iv) individuals with missing information on race or marital status (n = 81). After these exclusions, 1,071 node-positive MTC patients remained for final analysis from SEER database.

**Figure 1:**
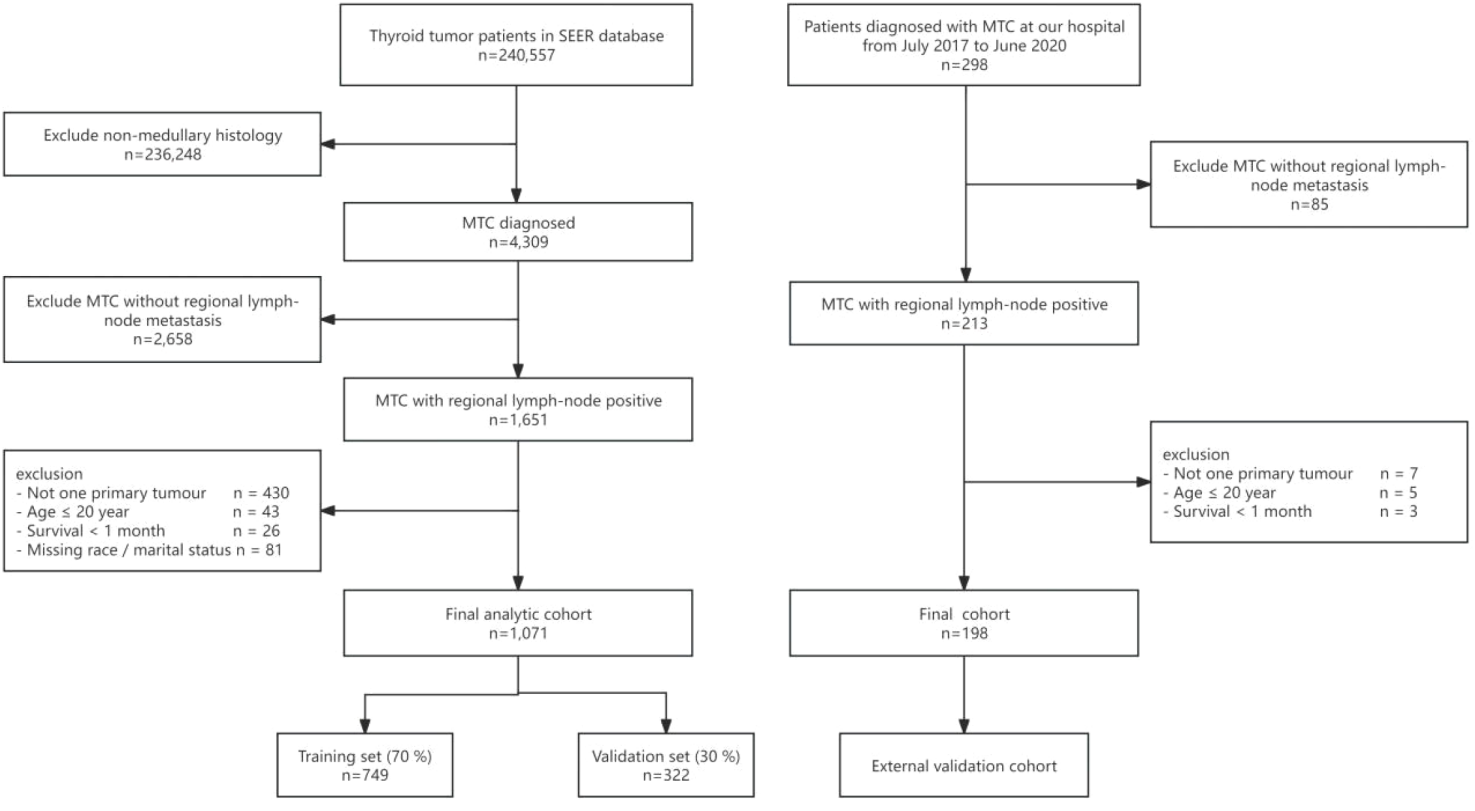
Flowchart of our study.

We identified 298 MTC patients diagnosed at Zibo Municipal Hospital between July 2017 and June 2020. After excluding (i) cases without pathologically confirmed regional lymph-node metastasis (n = 85), and (ii) patients with multiple primary malignancies (n = 7), age ≤ 20 years (n = 5), or survival < 1 month from diagnosis (n = 3), 198 node-positive MTC patients remained and constituted the final external validation cohort (**Figure 1**).

### Study covariates

The following baseline characteristics were extracted from SEER database and Zibo Municipal Hospital: age at diagnosis (< 60, 60-80, and ≥ 80 years), sex (female and male), and marital status (married and unmarried). Tumour-specific variables comprised the largest tumour diameter (< 4 cm, 4–6 cm, ≥ 6 cm, and unknown) and lymph node ratio (LNR) (<0.14, ≥0.14, and unknown) (11). Treatment-related factors included receipt of surgery (yes and no), radiotherapy (yes, no, and unknown), and chemotherapy (yes, no, and unknown). The SEER cohort captured both 3-year and 5-year overall survival (OS) and cancer-specific survival (CSS), whereas the external validation cohort recorded only 5-year OS.

For the mortality status, patients with missing survival status were excluded from analysis, as imputing survival outcomes could introduce significant bias. For other variables, missing cases were retained and coded as “Unknown”. No multiple imputation was performed for any variables.

### Feature selection and model development

We employed three feature selection strategies: Cox regression, Boruta algorithm, and recursive feature elimination (RFE).

First, univariable Cox proportional-hazards models were fitted to screen crude associations with mortality; variables exhibiting P < 0.05 were advanced to multivariable Cox analysis to identify independent prognostic factors, expressed as hazard ratios (HRs) with 95% confidence intervals (CIs). Second, Boruta algorithm identifies the most significant features in datasets by comparing each actual feature’s Z-score with its corresponding shadow feature’s Z-score. During this process, all actual features are replicated and shuffled to generate shadow features, which are then evaluated using a Random Forest model to obtain their Z-scores. These shadow feature Z-scores are derived from random permutations of the original features. If an actual feature consistently outperforms the maximum Z-score of its shadow feature across multiple independent tests, it is deemed “important” (marked in green) and classified as an acceptable variable. Conversely, if the actual feature’s Z-score does not significantly surpass that of its shadow feature, it is labelled as “unimportant” (marked in red) and classified as an unacceptable variable (20). Third, RFE trains an outer model on the complete feature set, removes the least important feature, and then repeats the “train-eliminate” cycle on the remaining features (21).

The SEER analytic cohort was randomly partitioned into training (70%) and testing (30%) sets after fixing a common random seed to ensure full reproducibility. After confirming that baseline characteristics were well balanced between the training and validation sets (P > 0.05), we trained five machine-learning algorithms on the training set and evaluated their performance in both the test set and the external validation cohort: extreme gradient boosting (XGBoost), light gradient boosting machine (LightGBM), random forest (RF), multilayer perceptron (MLP), and K-nearest neighbours (KNN) (22–24). **Supplementary Table 1** provides the hyperparameter settings of the models.

### Model evaluation

For each model, we computed seven global metrics: accuracy, area under the receiver operating characteristic (ROC) curve (AUC), F1-score, Matthews correlation coefficient (MCC), sensitivity, and specificity. Discrimination was quantified with ROC analysis; the AUC served as the primary global metric, and a higher AUC value indicates better performance of the machine learning model (25). Calibration plots showed that predicted probabilities were binned and plotted against observed event frequencies; perfect correspondence is represented by the 45° diagonal. Clinical utility was estimated with decision-curve analysis (DCA), which calculates the net benefit of a prediction model across a range of clinically relevant risk thresholds, thereby balancing true-positive findings against false-positive interventions. Model selection was based on a comprehensive performance evaluation across multiple metrics.

### Model explainability

Shapley Additive exPlanations (SHAP) values, derived from game theory, are used to interpret machine learning models. They reveal which features most significantly influence model predictions and how each feature affects the model’s output (26).

### Statistical analysis

Categorical variables were compared using the chi-square test and Fisher’s exact test, with Fisher’s exact test applied when any expected frequency was less than 5; otherwise, the chi-square test was used. Results were expressed as frequencies (n) and percentages (%). All analyses were performed with R software (version 4.1.2; R Foundation for Statistical Computing, Vienna, Austria). Model development, hyper-parameter tuning, and validation were carried out using the mlr3 package.

To facilitate clinicians, we have developed an online calculator based on predictive models. This calculator provides rapid predictive outcomes based on input patient characteristics.

## Results

### Patient characteristics

**Table 1** summarises the baseline characteristics of the 1,269 node-positive MTC patients stratified by data source. Overall, the SEER (n = 1,071) and external validation (n = 198) cohorts were well balanced: the median age distribution, sex, marital status, surgical resection, LNR, and chemotherapy use were comparable across groups (all P ≥ 0.05). A higher radiotherapy rate and a smaller tumor size were observed in the external validation cohort (P <0.05).

**Table 1:** Comparison of baseline characteristics between SEER cohort and external validation cohort in MTC patients with lymph-node metastasis.

Characteristics	Overall (N = 1,269)	SEER cohort (N = 1,071)	External validation cohort (N = 198)	P value
Age, n (%)				0.258
<60	790 (62.25)	666 (62.18)	124 (62.63)	
60-80	422 (33.25)	361 (33.71)	61 (30.81)	
≥80	57 (4.49)	44 (4.11)	13 (6.57)	
Sex, n (%)				0.082
Female	601 (47.36)	496 (46.31)	105 (53.03)	
Male	668 (52.64)	575 (53.69)	93 (46.97)	
Marriage, n (%)				0.654
Not married	473 (37.27)	402 (37.54)	71 (35.86)	
Married	796 (62.73)	669 (62.46)	127 (64.14)	
Surgery, n (%)				0.784
No	181 (14.26)	154 (14.38)	27 (13.64)	
Yes	1088 (85.74)	917 (85.62)	171 (86.36)	
Radiation, n (%)				0.025
No/unknown	952 (75.02)	816 (76.19)	136 (68.69)	
Yes	317 (24.98)	255 (23.81)	62 (31.31)	
Chemotherapy, n (%)				0.772
No/unknown	1081 (85.19)	911 (85.06)	170 (85.86)	
Yes	188 (14.81)	160 (14.94)	28 (14.14)	
Tumor size, n (%)				0.004
<4 cm	466 (36.72)	378 (35.29)	88 (44.44)	
4–6 cm	160 (12.61)	129 (12.04)	31 (15.66)	
≥6 cm	89 (7.01)	73 (6.82)	16 (8.08)	
Unknown	554 (43.66)	491 (45.85)	63 (31.82)	
LNR, n (%)				0.762
<0.14	173 (13.63)	147 (13.73)	26 (13.13)	
≥0.14	844 (66.51)	708 (66.11)	136 (68.69)	
Unknown	252 (19.86)	216 (20.17)	36 (18.18)	
5-year OS, n (%)				0.652
Alive	663 (68.00)	531 (68.34)	132 (66.67)	
Dead	312 (32.00)	246 (31.66)	66 (33.33)	

LNR, Lymph Node Ratio.

### Feature selection

**Figure 2:**
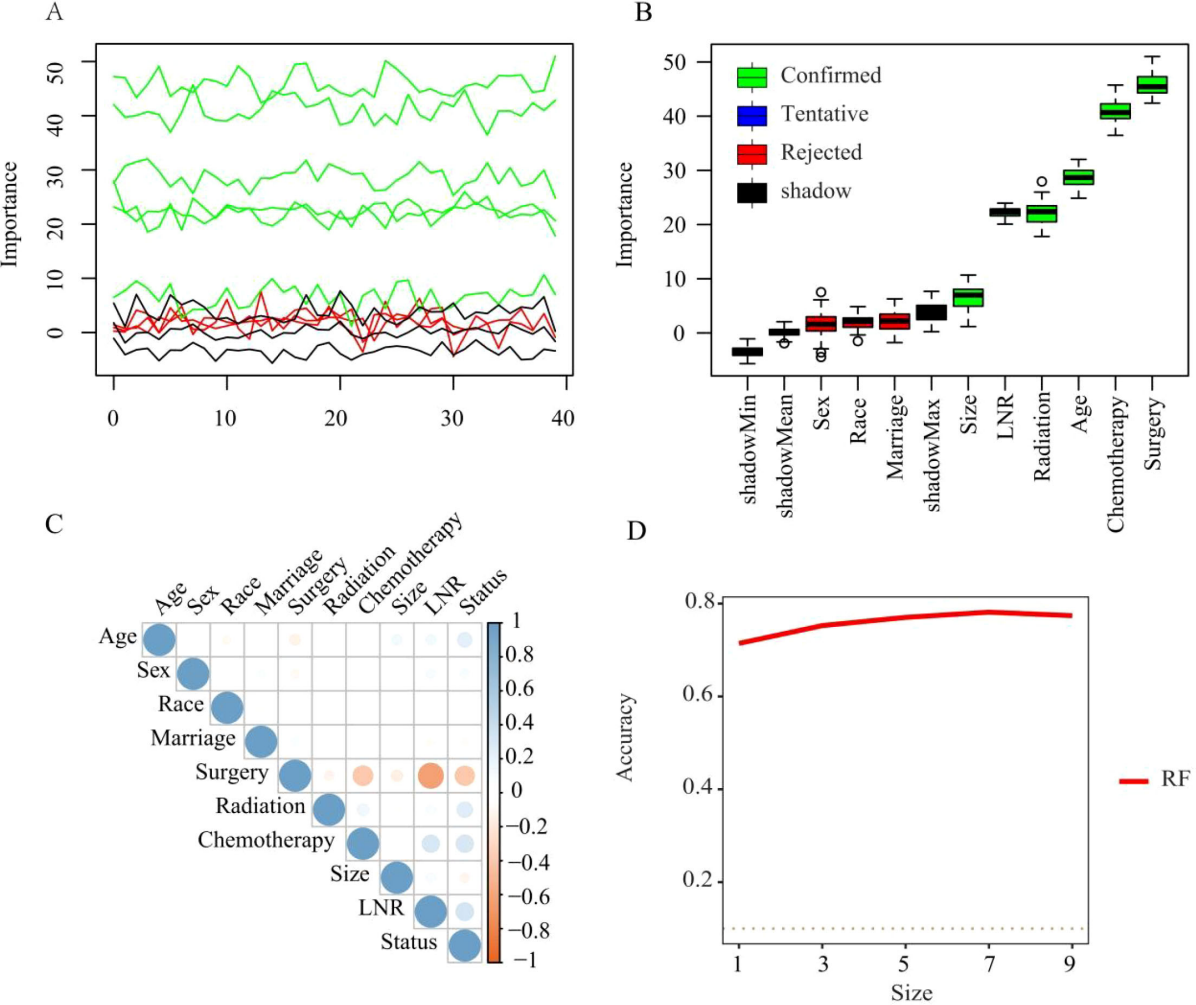
Variable selection among MTC patients with lymph-node metastases [**(A, B)** The figures show that the Boruta identified five confirmed feature variables. **(C)** The figure shows the Pearson correlation coefficient(r), with the colour gradient ranging from blue (r =–1) to red (r = +1), and white indicating r=0. **(D)** The figure shows that RFE achieved the highest prediction accuracy when seven feature variables were selected].

Univariable and multivariable Cox regression first identified seven candidate variables-age, marital status, surgery, radiotherapy, chemotherapy, tumour size, and LNR-as significantly associated with OS (**Table 2**). Application of Boruta algorithm retained six of these: age, surgery, radiotherapy, chemotherapy, tumour size, and LNR (**Figures 2A, B**), while RFE achieved maximal accuracy when the seven variables were retained (**Figure 2D**). Inter-correlation among the final predictors was low (**Figure 2C**), supporting their independence and collective inclusion in the subsequent machine-learning models.

**Table 2:** Univariate and multivariate Cox regression analyses of overall survival in MTC patients with lymph-node metastases.

Characteristics	Univariate		Multivariate	
	HR (95%CI)	*P*	HR (95%CI)	*P*
Age				
<60	1.00		1.00	
60-80	2.20 (1.76, 2.74)	<.001	2.00 (1.59, 2.51)	<.001
≥80	5.90 (4.04, 8.60)	<.001	4.26 (2.90, 6.26)	<.001
Sex				
Female	1.00		1.00	
Male	1.48 (1.20, 1.83)	<.001	1.12 (0.89, 1.40)	0.326
Race				
White	1.00			
Black	0.98 (0.68, 1.40)	0.909		
Other	0.60 (0.36, 1.01)	0.056		
Marriage				
Not married	1.00		1.00	
Married	0.79(0.64, 0.97)	0.025	0.79 (0.69, 0.99)	0.039
Surgery				
No	1.00		1.00	
Yes	0.16 (0.12, 0.20)	<.001	0.42 (0.27, 0.63)	<.001
Radiation				
No/unknown	1.00		1.00	
Yes	2.24 (1.81, 2.77)	<.001	1.48 (1.18, 1.84)	<.001
Chemotherapy				
No/unknown	1.00		1.00	
Yes	4.18 (3.32, 5.26)	<.001	1.85 (1.41, 2.44)	<.001
Tumor size				
<4 cm	1.00		1.00	
4–6 cm	1.68 (1.25, 2.26)	<.001	1.39 (1.02, 1.89)	0.035
≥6 cm	3.06 (2.19, 4.26)	<.001	1.70 (1.20, 2.41)	0.003
Unknown	1.60 (1.22, 2.09)	<.001	1.12 (0.85, 1.47)	0.414
LNR				
0.14	1.00		1.00	
≥0.14	1.57 (1.04, 2.36)	0.031	1.40 (0.92, 2.11)	0.113
Unknown	7.25 (4.77, 11.01)	<.001	2.32 (1.36, 3.95)	0.002

### Machine-learning model evaluation

**Figure 3:**
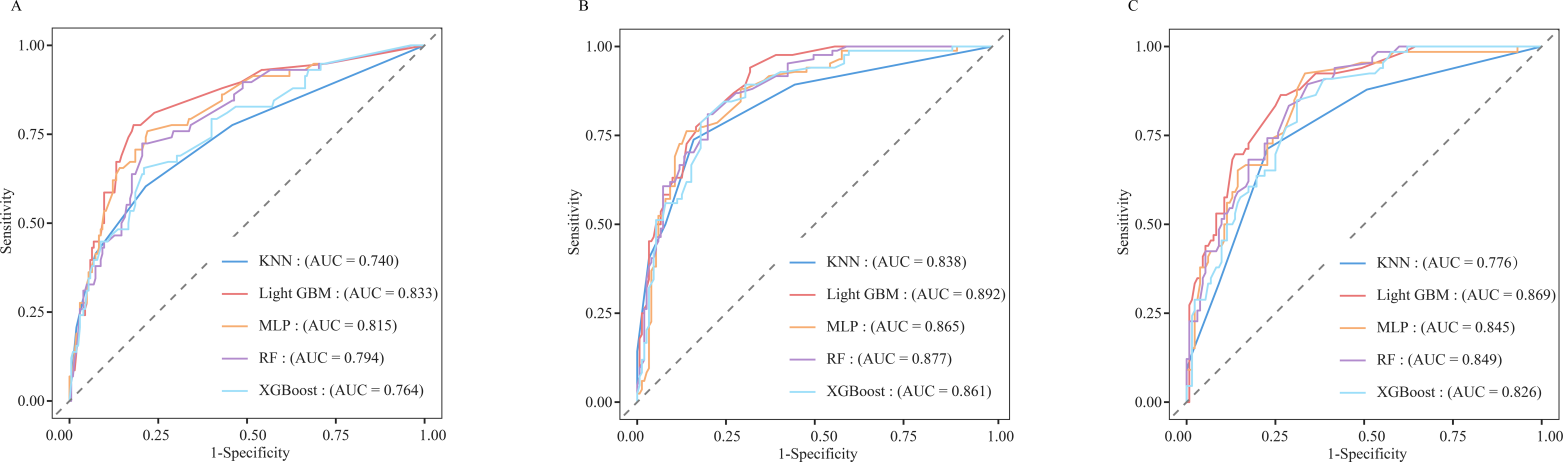
ROC curves of five different machine learning models [**(A)**: 3-year SEER cohort test set; **(B)**: 5-year SEER cohort test set; **(C)**: 5-year external validation cohort; RF, Random Forest; KNN, k-Nearest Neighbours; XGBoost, Xtreme Gradient Boosting; MLP, Multilayer Perceptron; LightGBM, Light Gradient Boosting Machine].

**Figure 4:**
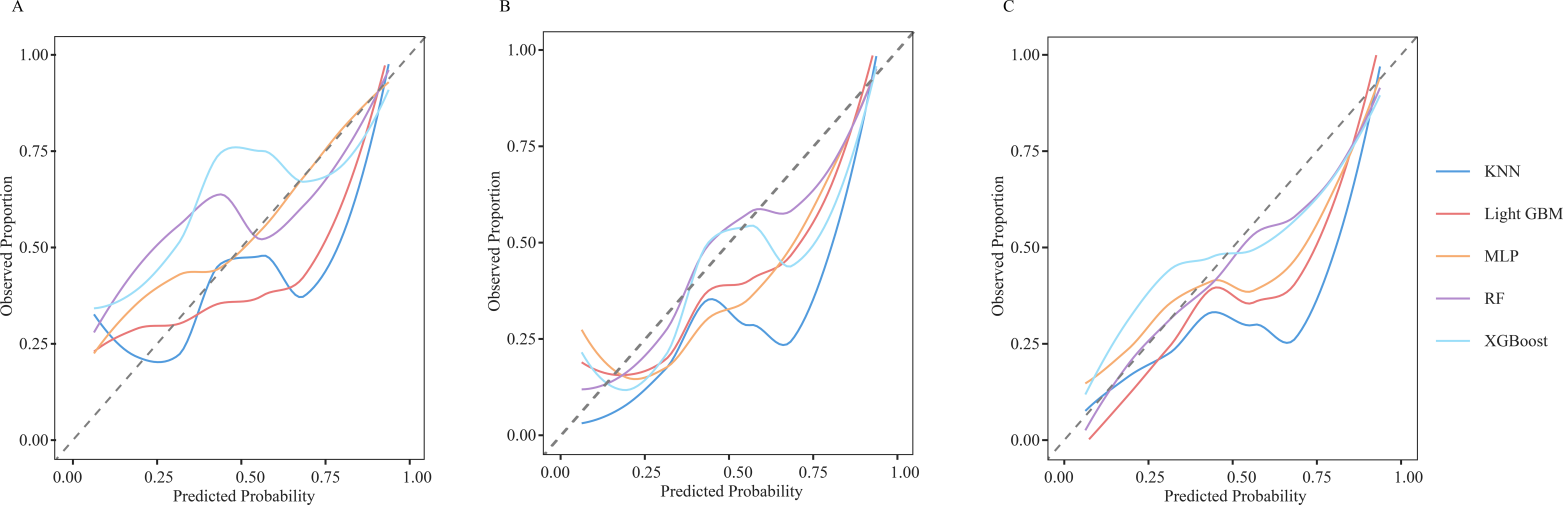
Calibration curves of five different machine learning models [**(A)** 3-year SEER cohort test set; **(B)** 5-year SEER cohort test set; **(C)** 5-year external validation cohort; RF, Random Forest; KNN, k-Nearest Neighbours; XGBoost, Xtreme Gradient Boosting; MLP, Multilayer Perceptron; LightGBM, Light Gradient Boosting Machine].

**Figure 5:**
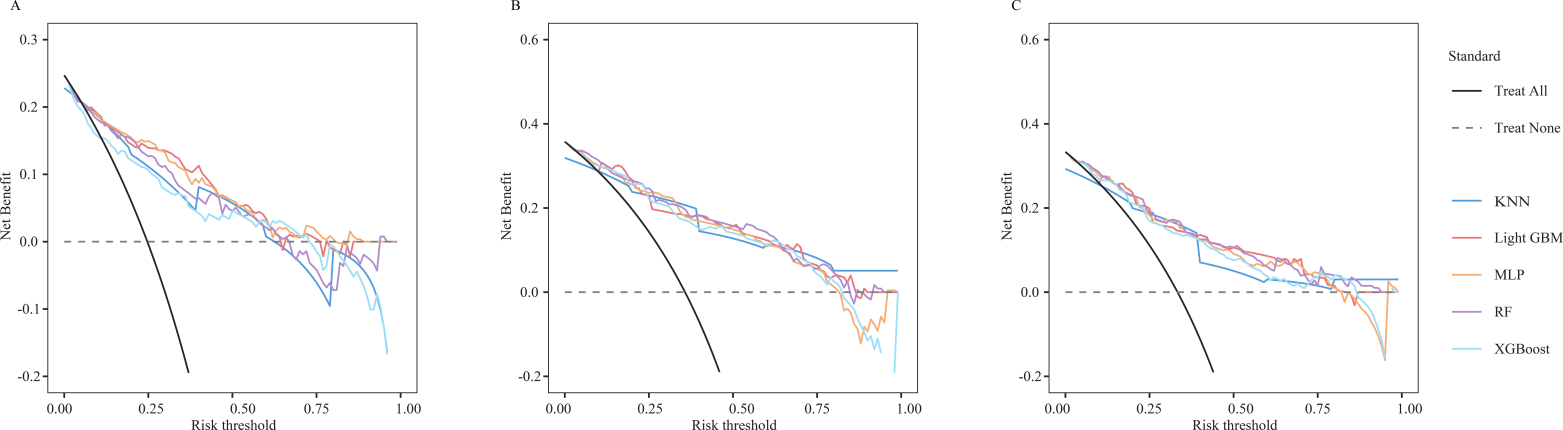
DCA curves of five different machine learning models [**(A)** 3-year SEER cohort test set; **(B)** 5-year SEER cohort test set; **(C)** 5-year external validation cohort; RF, Random Forest; KNN, k-Nearest Neighbours; XGBoost, Xtreme Gradient Boosting; MLP, Multilayer Perceptron; LightGBM, Light Gradient Boosting Machine).

After identifying the six candidate variables, we used 3- and 5-year OS as the endpoint in the SEER training set to train the five candidate algorithms: RF, KNN, XGBoost, MLP, and LightGBM. The models were then compared in the SEER test set (endpoints: 3- and 5-year OS) and in the external validation cohort (endpoint: 5-year OS). LightGBM delivered the highest accuracy and AUC (SEER cohort test set, 3-year OS: Accuracy = 0.821, AUC = 0.833; 5-year OS: Accuracy = 0.800, AUC = 0.892; External validation cohort, Accuracy = 0.773, AUC = 0.869; **Table 3**). ROC analysis also confirmed that LightGBM consistently outperformed the other four learning methods in distinguishing mortality rates at any given time point (**Figure 3**). Calibration plots revealed that its predicted probabilities lay closest to the ideal 45° reference line at both three and five years, signifying optimal agreement between expected and observed outcomes (**Figure 4**). DCA further demonstrated that, across the entire clinically plausible range of risk thresholds, LightGBM conferred the highest net benefit (**Figure 5**). Similar results were obtained when predicting CSS in SEER cohort (**Supplementary Table 2**; **Supplementary Figure 1**).

**Table 3:** Performance metrics of the five machine-learning models in the SEER cohort test set and the external validation cohort.

Target	Model	Accuracy	AUC	F1-Score	MCC	Sensitivity	Specificity
SEER cohort test set							
3-years OS	RF	0.798 (0.778, 0.818)	0.794 (0.774, 0.814)	0.485 (0.465, 0.505)	0.367 (0.347, 0.387)	0.431 (0.411, 0.451)	0.902 (0.882, 0.922)
	KNN	0.814 (0.794, 0.834)	0.740 (0.720, 0.760)	0.495 (0.475, 0.515)	0.397 (0.377, 0.417)	0.414 (0.394, 0.434)	0.927 (0.907, 0.947)
	XGBoost	0.791 (0.771, 0.811)	0.764 (0.744, 0.784)	0.495 (0.475, 0.515)	0.365 (0.345, 0.385)	0.466 (0.446, 0.486)	0.883 (0.863, 0.903)
	MLP	0.814 (0.794, 0.834)	0.815 (0.795, 0.835)	0.533 (0.513, 0.553)	0.422 (0.402, 0.442)	0.483 (0.463, 0.503)	0.907 (0.887, 0.927)
	LightGBM	0.821 (0.801, 0.841)	0.833 (0.813, 0.853)	0.505 (0.485, 0.525)	0.418 (0.398, 0.438)	0.414 (0.394, 0.434)	0.937 (0.917, 0.957)
5-years OS	RF	0.800 (0.780, 0.820)	0.877 (0.857, 0.897)	0.685 (0.665, 0.705)	0.551 (0.531, 0.571)	0.607 (0.587, 0.627)	0.907 (0.887, 0.927)
	KNN	0.770 (0.750, 0.790)	0.838 (0.818, 0.858)	0.609 (0.589, 0.629)	0.479 (0.459, 0.499)	0.500 (0.480, 0.520)	0.921 (0.901, 0.941)
	XGBoost	0.783 (0.763, 0.803)	0.861 (0.841, 0.881)	0.633 (0.613, 0.653)	0.510 (0.490, 0.530)	0.524 (0.504, 0.544)	0.927 (0.907, 0.947)
	MLP	0.791 (0.771, 0.811)	0.865 (0.845, 0.885)	0.657 (0.637, 0.677)	0.531 (0.511, 0.551)	0.560 (0.540, 0.580)	0.921 (0.901, 0.941)
	LightGBM	0.800 (0.780, 0.820)	0.892 (0.872, 0.912)	0.676 (0.656, 0.696)	0.551 (0.531, 0.571)	0.583 (0.563, 0.603)	0.921 (0.901, 0.941)
External validation cohort							
5-years OS	RF	0.773 (0.753, 0.793)	0.849 (0.829, 0.869)	0.602 (0.582, 0.622)	0.462 (0.442, 0.482)	0.515 (0.495, 0.535)	0.902 (0.882, 0.922)
	KNN	0.717 (0.697, 0.737)	0.776 (0.756, 0.796)	0.440 (0.420, 0.460)	0.303 (0.283, 0.323)	0.333 (0.313, 0.353)	0.909 (0.889, 0.929)
	XGBoost	0.742 (0.722, 0.762)	0.826 (0.806, 0.846)	0.541 (0.521, 0.561)	0.383 (0.363, 0.403)	0.455 (0.435, 0.475)	0.886 (0.866, 0.906)
	MLP	0.758 (0.738, 0.778)	0.845 (0.825, 0.865)	0.571 (0.551, 0.591)	0.423 (0.403, 0.443)	0.485 (0.465, 0.505)	0.894 (0.874, 0.914)
	LightGBM	0.773 (0.753, 0.793)	0.869 (0.849, 0.889)	0.571 (0.551, 0.591)	0.458 (0.438, 0.478)	0.455 (0.435, 0.475)	0.932 (0.912, 0.952)

AUC, Area Under the Receiver Operating Characteristic Curve; F1 = 2 × (Precision × Recall) / (Precision + Recall); MCC, Matthews Correlation Coefficient; RF, Random Forest; KNN, k-Nearest Neighbors; XGBoost, Xtreme Gradient Boosting; MLP, Multilayer Perceptron; LightGBM, Light Gradient Boosting Machine.

### Model interpretability

**Figure 6:**
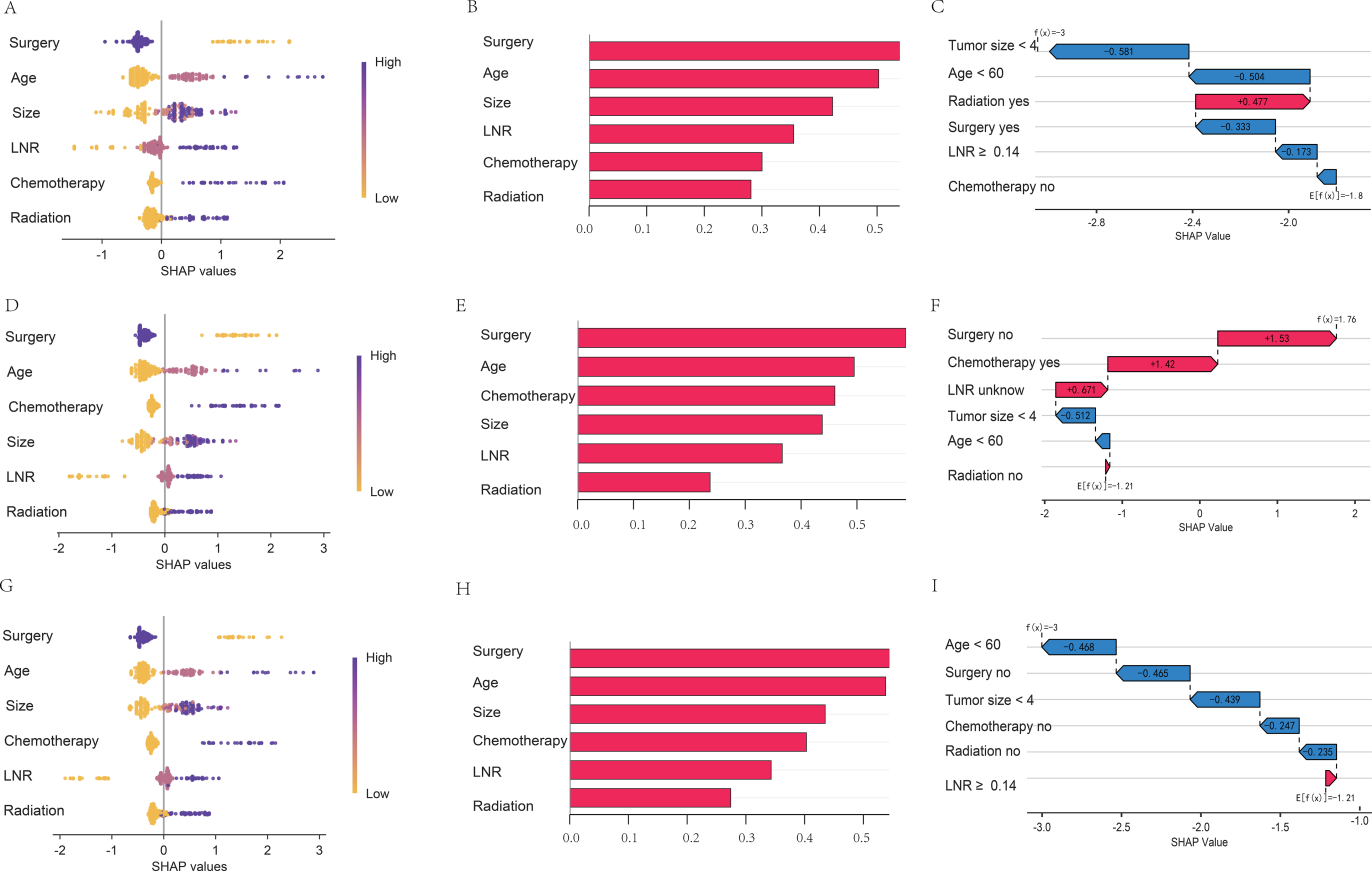
Feature importance ranking in the OS prediction model based on LightGBM algorithm. [**(A-C)** 3-year SEER cohort test set; **(D-F)** 5-year SEER cohort test set; **(G-I)** 5-year external validation cohort; In the SHAP beeswarm plot, purple indicates higher values and yellow indicates lower ones; a SHAP value greater than 0 on the x-axis signifies increased mortality risk, while a value less than 0 indicates decreased mortality risk; In the SHAP bar curve, the x-axis represents the SHAP value’s impact on the model’s output, the higher the value of the x-axis, the greater the impact on the model; The SHAP waterfall plot illustrates the decomposition of the prediction for an individual sample, where red bars represent features that increase mortality risk (positive values, pointing right), blue bars represent features that decrease mortality risk (negative values, pointing left), and the bar length indicates the magnitude of contribution].

SHAP analysis revealed that, in both the SEER cohort and the external validation cohort, the absence of surgical treatment exerted the strongest adverse effect on OS prediction for MTC, resulting in elevated individual risk assessment values. Advanced age, larger tumour size, higher LNR, and receipt of chemoradiotherapy likewise increased death probability (**Figure 6**). **Figures 6C, F, I** illustrate this cumulative effect for the first patient in the cohort, showing how each clinical variable sequentially updates the model’s baseline output to yield a subject-specific probability of death. Similar results were obtained when predicting CSS (**Supplementary Figure 2**).

### Network calculator building

**Figure 7:**
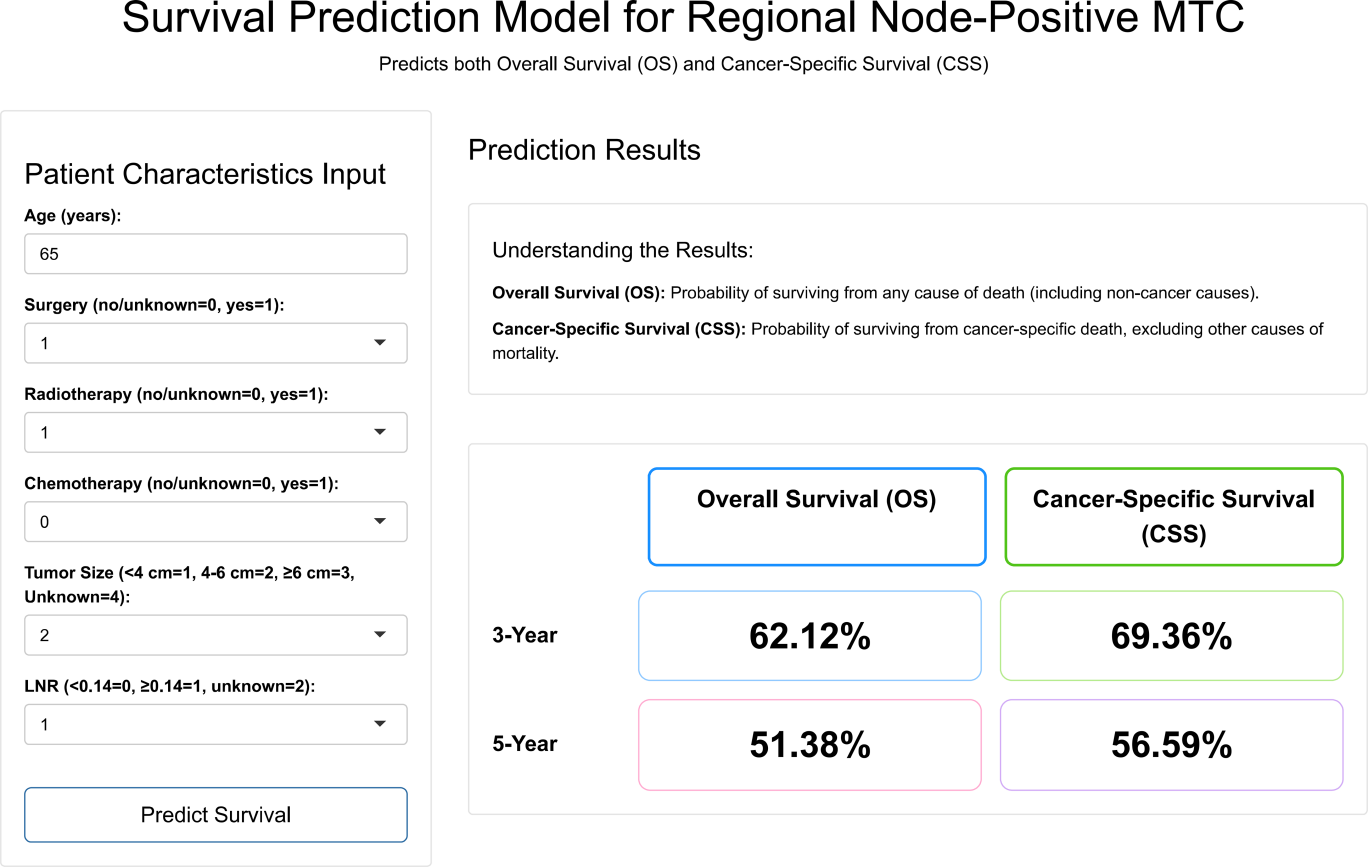
Screenshot of the online prediction calculator.

Finally, an online calculator was developed based on the results of LightGBM model (http://122.51.24.245:3838/MTC/). The interface is shown in **Figure 7**. Clinicians can input the six predictive factor indices of lymph-node-positive MTC patients and instantly generate 3- and 5-year OS and CSS predictions.

## Discussion

MTC is a comparatively rare neuroendocrine malignancy, accounting for only 4% of all thyroid tumours yet responsible for approximately 13% of thyroid-cancer-related deaths (27, 28). Roughly one-quarter of cases are hereditary, whereas 75% are sporadic; among the latter, approximately 60% harbour somatic RET mutations, providing a rational target for RET-selective inhibitors such as selpercatinib and pralsetinib (29). Unlike differentiated thyroid cancers, MTC neither concentrates radioiodine nor responds to TSH suppression, so curative-intent treatment has traditionally relied on surgical resection; nevertheless, outcomes for advanced disease remain poor (30). Consequently, detailed delineation of the determinants of survival and the development of precise prognostic tools are essential to refine therapeutic strategies and improve the quality of life of patients with MTC.

Using retrospective cohorts from the U.S. SEER database and a Chinese hospital, we systematically evaluated multidimensional clinical determinants of survival in lymph-node-positive MTC patients and constructed a machine-learning-based prognostic assessment framework. Consistent with previous reports, surgical resection emerged as the dominant predictor of both 3- and 5-year mortality, underscoring its central role in the management of locoregionally advanced disease (30). Age ranked second or third in feature importance across the two time horizons; immunosenescence and age-related alterations in the tumour micro-environment are well-recognised mechanisms that enhance tumour aggressiveness and metastatic potential, explaining the higher mortality observed among elderly MTC patients (30, 31). Tumour size was another key contributor to the risk of death, corroborating earlier data demonstrating that larger primary tumours are associated with increased recurrence rates (32). Higher LNR is associated with increased mortality risk. Rozenblat et al. demonstrated in a multi-center cohort that LNR was an independent predictor of survival, outperforming the absolute number of positive nodes (12). Chemotherapy and radiotherapy should be interpreted not as independent risk factors for mortality, but rather as clinical proxies for advanced disease state in our study. In the SHAP beeswarm analysis, these therapies push predictions toward higher mortality not because the treatments themselves worsen survival, but because they are clinical proxies for situations such as: gross residual disease, extrathyroidal extension, unresectable or metastatic disease (33, 34). This finding is consistent with previous studies on MTC patients from SEER database (31, 35).

Machine learning has been increasingly applied to thyroid cancer prognostication (36–38). Recently, Fang et al. employed machine learning to identify immune cell-related gene signatures for thyroid cancer stratification, provide novel biomarkers for the precise diagnosis and individualized treatment of thyroid cancer (39). Park and Lee developed a Random Forest model for papillary thyroid carcinoma recurrence prediction using clinico-pathologic factors (13). Liu et al. combined radiopathomic features for risk stratification (14). Lee and Park provided a comprehensive review of ML applications in thyroid disease, noting that most studies focused on DTC rather than MTC (15). Specifically for MTC, Guo et al. developed machine learning models to predict distant metastasis using SEER data (16). To our knowledge, this is the first machine learning study specifically targeting lymph-node-positive MTC, a high-risk population with disproportionate mortality burden that has been previously understudied. So, we constructed five prognostic frameworks to forecast 3- and 5-year mortality in node-positive MTC and evaluated six complementary metrics-accuracy, MCC, sensitivity, specificity, F1-score, and AUC-across five algorithms. LightGBM consistently achieved the highest performance at both horizons, an advantage attributable to its gradient-boosting decision-tree architecture that efficiently captures complex non-linear relationships and high-order interactions among clinical variables (40). Moreover, this study represents the first systematic head-to-head comparison of multiple machine-learning algorithms for survival prediction in MTC, establishing an optimal model that furnishes clinicians with a more accurate and individualized risk-assessment tool.

Our binary “surgery” variable includes total thyroidectomy, thyroidectomy, and subtotal or near-total thyroidectomy. Incomplete surgical approaches are generally considered to be insufficient for the treatment of lymph node-positive MTC. However, several considerations support the stability of our primary model. First, the clinical management of MTC follows an aggressive surgical paradigm where total thyroidectomy with compartment-oriented lymphadenectomy is the established standard of care; subtotal or incomplete resections are rarely performed intentionally and typically represent uncommon scenarios. Second, the SHAP analysis consistently identified “absence of surgery” as the strongest adverse predictor across all time horizons, suggesting that the model primarily distinguishes between surgical intervention versus no intervention. Third, the external validation in our Chinese cohort demonstrated excellent model performance, further supporting the generalizability of the binary classification approach.

The 2022 International MTC Grading System identifies high-grade features (mitotic count ≥5 per 2 mm², Ki-67 proliferation index ≥5%, and tumor necrosis) as independent prognostic factors for disease-specific survival (10). While our machine learning model incorporates clinicopathological variables available in SEER (age, tumor size, lymph node status), it does not include these histologic grading parameters due to database limitations. Nevertheless, our model’s identification of tumor size and surgical resection status as dominant predictors aligns conceptually with the grading system’s emphasis on tumor biology and extent of disease. Future studies will integrate histological grading into machine learning models, which may further improve the accuracy of prognostic prediction.

The RET M918T mutation is present in approximately 80% of sporadic MTC harboring somatic RET alterations and is strongly associated with aggressive disease biology, including larger tumor size, advanced AJCC stage, and lymph node metastasis (41–43). The current model-although biomarker-agnostic-may indirectly capture high-risk molecular phenotypes through these macroscopic clinical features. The predicted OS and CSS probabilities generated by our model should be interpreted as historical baselines, derived from SEER cohorts largely treated prior to widespread access to modern RET-selective inhibitors. In 2020, the US FDA approved selpercatinib and pralsetinib for RET-mutant MTC based on the LIBRETTO-001 and ARROW trials, which demonstrated objective response rates of approximately 73% and 71%, respectively, with durable disease control in patients with progressive disease (29, 44). For patients diagnosed today who receive selpercatinib or pralsetinib, survival outcomes may exceed these model predictions. In this context, the model may serve as a useful tool to identify patients who should undergo prompt germline and somatic RET testing.

Extranodal extension (ENE) refers to the invasion of metastatic cancer into surrounding soft tissues through the lymph node capsule (45–47). Although not formally incorporated into the current AJCC T-stage for MTC, ENE has been consistently associated with increased local recurrence rates and mortality in differentiated thyroid cancer and is increasingly recognized as an adverse prognostic factor in MTC (48–51). The absence of ENE data in SEER database and external validation represents a significant limitation of our model, as this variable would likely enhance risk stratification.

The present study has three main limitations. First, despite the large sample size provided by SEER database, its retrospective design inherently introduces the possibility of selection bias. Detailed pathological parameters are not comprehensively recorded, potentially leading to the omission of important prognostic factors. Second, although external validation was performed, the model should be further evaluated in cohorts from additional institutions or geographic regions to enhance its generalizability and reliability for clinical practice. Third, for node-positive MTC patients, any degree of surgery less than total thyroidectomy with comprehensive lymphadenectomy is considered inadequate. However, due to data limitations, our study did not differentiate this specific surgical population; future prospective studies should consider analyzing these surgery patients separately. Finally, an important limitation of this study is the absence of calcitonin and carcinoembryonic antigen (CEA) doubling times, which are standard biomarkers for MTC surveillance and prognostication. While these data are unavailable in SEER database, their exclusion limits the clinical depth of our predictive model. Future prospective studies incorporating these dynamic biomarkers may enhance prognostic accuracy and better guide individualized treatment decisions.

## Conclusion

In this large, externally validated cohort of lymph-node-positive MTC patients, LightGBM model delivered superior discrimination, calibration, and clinical net benefit for predicting 3- and 5-year OS and CSS. The model’s interpretability underscores surgery and age as the dominant modifiable and non-modifiable drivers of survival, respectively.

## Data Availability

The raw data supporting the conclusions of this article will be made available by the authors, without undue reservation.
