# Supplementary material for: Machine-learning prediction of 3- and 5-year mortality in lymph-node-positive medullary thyroid carcinoma: a study based on the SEER database and external validation in a Chinese cohort
Source: Front Oncol. 2026 Apr 14;16:1798296. doi: 10.3389/fonc.2026.1798296 (PMC13120900; doi:10.3389/fonc.2026.1798296)
Supplement: Supplementary file 1 [file DataSheet1.docx]

**Supplement tables and figures**

**Table S1. Hyperparameter configuration for LightGBM and XGBoost with theoretical justification;**

**Table S2. Performance metrics of the five machine-learning models in the SEER cohort test set;**

**Figure S1. Performance evaluation of machine learning models for CSS prediction in the SEER cohort test set;**

**Figure S2. Feature importance ranking in the CSS prediction model based on the LightGBM algorithm.**

| **Table S1. Hyperparameter configuration for LightGBM and XGBoost with theoretical justification** | | | |
| --- | --- | --- | --- |
| Models | Parameter | Value | Illustration |
| LightGBM | n_estimators | 200 | Adapt to small samples and 5 core features |
|  | learning_rate | 0.1 | Balancing Fitting Speed and Performance |
|  | max_depth | 5 | Limiting tree complexity to improve the generalization of external validation sets |
|  | num_leaves | 32 | Satisfies num_leaves < 2^max_depth, suitable for binary mortality prediction tasks |
|  | min_child_samples | 10 | Curbing extreme cases from overfitting |
| XGBoost | n_estimators | 200 | Consistent with LightGBM |
|  | learning_rate | 0.1 | Consistent with LightGBM |
|  | max_depth | 5 | Limiting tree complexity |
|  | min_child_weight | 3 | Curbing extreme cases from overfitting |
|  | gamma | 0.1 | Set weak split loss to avoid meaningless node splits and optimize binary classification prediction performance |
| XGBoost: Xtreme Gradient Boosting; LightGBM: Light Gradient Boosting Machine. | | | |

| **Table S2. Performance metrics of the five machine-learning models in the SEER cohort test set** | | | | | | | |
| --- | --- | --- | --- | --- | --- | --- | --- |
| Target | Model | Accuracy | AUC | F1-Score | MCC | Sensitivity | Specificity |
| Test set in SEER cohort | | | | | | | |
| 3-years CSS | RF | 0.817 (0.797, 0.837) | 0.833 (0.813, 0.853) | 0.540 (0.520, 0.560) | 0.433 (0.413, 0.453) | 0.482 (0.462, 0.502) | 0.913 (0.893, 0.933) |
|  | KNN | 0.849 (0.829, 0.869) | 0.763 (0.743, 0.783) | 0.578 (0.558, 0.598) | 0.515 (0.495, 0.535) | 0.464 (0.444, 0.484) | 0.959 (0.939, 0.979) |
|  | XGBoost | 0.810 (0.790, 0.830) | 0.788 (0.768, 0.808) | 0.510 (0.490, 0.530) | 0.401 (0.381, 0.421) | 0.446 (0.426, 0.466) | 0.913 (0.893, 0.933) |
|  | MLP | 0.829 (0.809, 0.849) | 0.801 (0.781, 0.821) | 0.574 (0.554, 0.594) | 0.474 (0.454, 0.494) | 0.518 (0.498, 0.538) | 0.918 (0.898, 0.938) |
|  | LightGBM | 0.833 (0.813, 0.853) | 0.850 (0.830, 0.870) | 0.533 (0.513, 0.553) | 0.459 (0.439, 0.479) | 0.429 (0.409, 0.449) | 0.949 (0.929, 0.969) |
| 5-years CSS | RF | 0.797 (0.777, 0.817) | 0.832 (0.812, 0.852) | 0.646 (0.626, 0.666) | 0.513 (0.493, 0.533) | 0.577 (0.557, 0.597) | 0.901 (0.881, 0.921) |
|  | KNN | 0.784 (0.764, 0.804) | 0.807 (0.787, 0.827) | 0.564 (0.544, 0.584) | 0.470 (0.450, 0.490) | 0.437 (0.417, 0.457) | 0.947 (0.927, 0.967) |
|  | XGBoost | 0.802 (0.782, 0.822) | 0.843 (0.823, 0.863) | 0.656 (0.636, 0.676) | 0.526 (0.506, 0.546) | 0.592 (0.572, 0.612) | 0.901 (0.881, 0.921) |
|  | MLP | 0.806 (0.786, 0.826) | 0.841 (0.821, 0.861) | 0.639 (0.619, 0.659) | 0.531 (0.511, 0.551) | 0.535 (0.515, 0.555) | 0.934 (0.914, 0.954) |
|  | LightGBM | 0.797 (0.777, 0.817) | 0.852 (0.832, 0.872) | 0.646 (0.626, 0.666) | 0.513 (0.493, 0.533) | 0.577 (0.557, 0.597) | 0.901 (0.881, 0.921) |
| AUC: Area Under the Receiver Operating Characteristic Curve; F1 = 2 × (Precision × Recall) / (Precision + Recall); MCC, Matthews Correlation Coefficient; RF: Random Forest; KNN: k-Nearest Neighbors; XGBoost: Xtreme Gradient Boosting; MLP: Multilayer Perceptron; LightGBM: Light Gradient Boosting Machine; CSS, Cancer-Specific Survival. | | | | | | | |


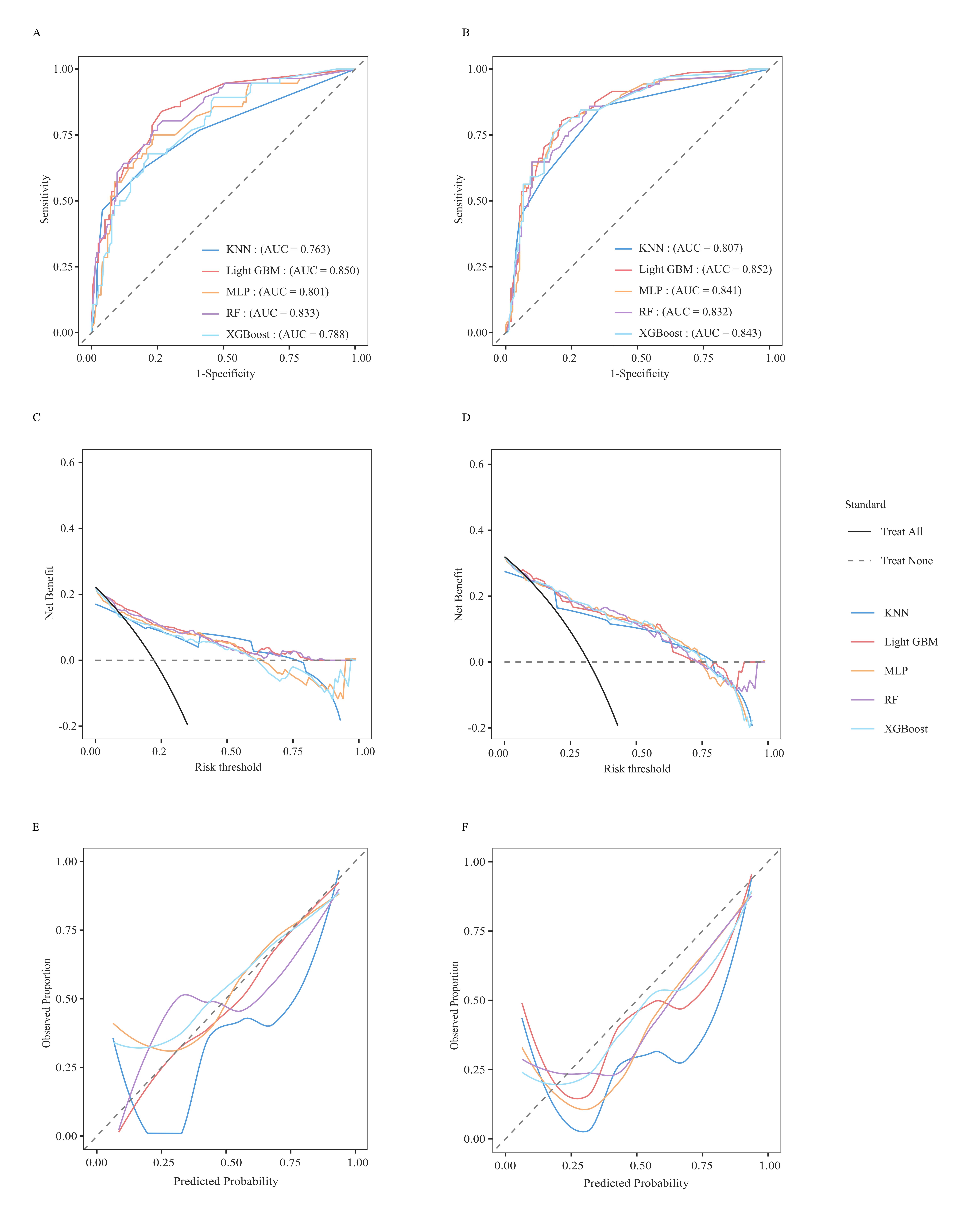


**Figure S1. Performance evaluation of machine learning models for CSS prediction in the SEER cohort test set.** ROC curves for 3-year (A) and 5-year (B) CSS; DCA for 3-year (C) and 5-year (D) CSS; Calibration curves for 3-year (E) and 5-year (F) CSS.


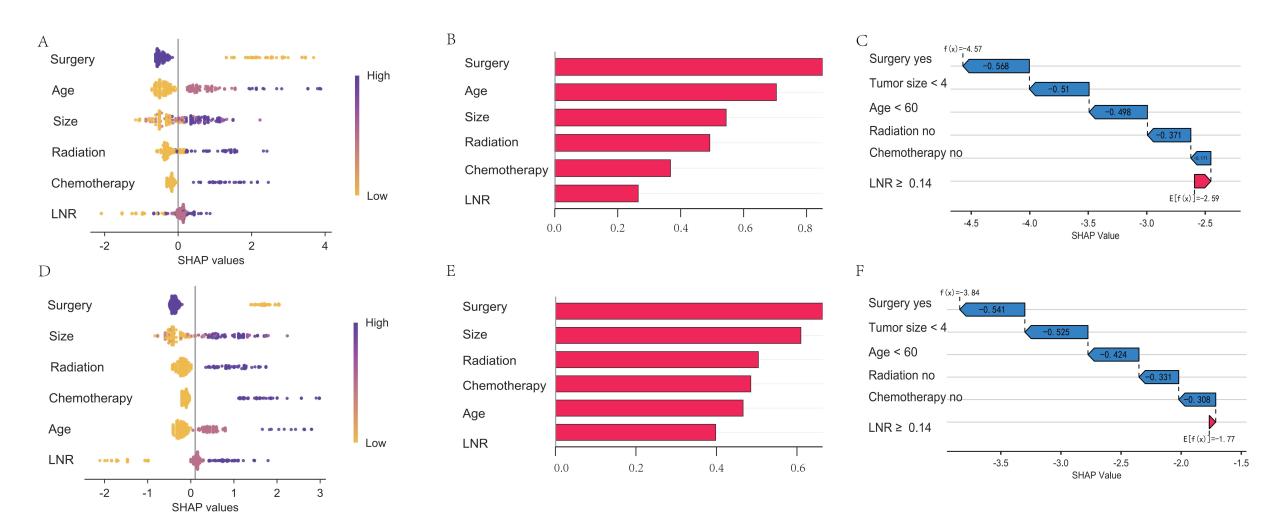


**Figure S2. Feature importance ranking in the CSS prediction model based on the LightGBM algorithm.** A, B & C: 3-year CSS of SEER cohort test set; D, E&F: 5-year CSS of SEER cohort test set; In the SHAP beeswarm plot, purple indicates higher eigenvalues and yellow indicates lower ones; a Shap value greater than 0 on the x-axis signifies increased mortality risk, while a value less than 0 indicates decreased mortality risk; In the SHAP bar curve, the x-axis represents the SHAP value’s impact on the model’s output, the higher the value of the x-axis, the greater the impact on the model; The SHAP waterfall plot illustrates the decomposition of the prediction for an individual sample, where red bars represent features that increase mortality risk (positive values, pointing right), blue bars represent features that decrease mortality risk (negative values, pointing left), and the bar length indicates the magnitude of contribution.
